# A Lipid Mediator Hepoxilin A3 Is a Natural Inducer of Neutrophil Extracellular Traps in Human Neutrophils

**DOI:** 10.1155/2015/520871

**Published:** 2015-02-16

**Authors:** David N. Douda, Hartmut Grasemann, Cecil Pace-Asciak, Nades Palaniyar

**Affiliations:** ^1^Lung Innate Immunity Research Laboratory, Program in Physiology & Experimental Medicine, The Hospital for Sick Children Research Institute, Toronto, ON, Canada M5G 0A4; ^2^Department of Laboratory Medicine & Pathobiology, University of Toronto, Toronto, ON, Canada M5G 0A4; ^3^Pulmonary & Critical Care Medicine Division, Brigham & Women's Hospital, Harvard Medical School, Boston, MA 02115, USA; ^4^Division of Respiratory Medicine, Department of Paediatrics, The Hospital for Sick Children, Toronto, ON, Canada M5G 1X8; ^5^Institute of Medical Sciences, University of Toronto, Toronto, ON, Canada M5S 1A8; ^6^Department of Pharmacology and Toxicology, University of Toronto, Toronto, ON, Canada M5S 1A8

## Abstract

Pulmonary exacerbations in cystic fibrosis airways are accompanied by inflammation, neutrophilia, and mucous thickening. Cystic fibrosis sputum contains a large amount of uncleared DNA contributed by neutrophil extracellular trap (NET) formation from neutrophils. The exact mechanisms of the induction of NETosis in cystic fibrosis airways remain unclear, especially in uninfected lungs of patients with early cystic fibrosis lung disease. Here we show that Hepoxilin A3, a proinflammatory eicosanoid, and the synthetic analog of Hepoxilin B3, PBT-3, directly induce NETosis in human neutrophils. Furthermore, we show that Hepoxilin A3-mediated NETosis is NADPH-oxidase-dependent at lower doses of Hepoxilin A3, while it is NADPH-oxidase-independent at higher doses. Together, these results demonstrate that Hepoxilin A3 is a previously unrecognized inducer of NETosis in cystic fibrosis lungs and may represent a new therapeutic target for treating cystic fibrosis and other inflammatory lung diseases.

## 1. Introduction

In infected and inflamed tissues, recruited neutrophils release neutrophil extracellular traps (NETs) [1]. NETs are made of DNA, and the elaborate strings of DNA are decorated with histones and antimicrobial proteins and peptides [2]. While it was originally thought that NETs are an effective method by which neutrophils trap and kill bacteria [3], a number of studies now show the deleterious “side-effects” of NETs, especially when overproduced [4].

It is now known that NETs directly cause host cell death [5] and are directly linked to the pathogenesis of a number of lung disorders including transfusion related acute lung injury (TRALI) [6, 7], ventilator induced lung injury (VILI) [8], pneumonia [9, 10], and cystic fibrosis (CF) [11]. However, the precise mechanisms leading to the excess neutrophil recruitment, activation, and NET production are not clearly understood. Thus, the negative impact of NETs demonstrated in a variety of inflammatory disorders illustrates the need to better understand NETosis and its signaling pathways and physiological mechanisms in regulating NETosis in health.

Hepoxilin A3 (HxA3) is a hydroxyepoxide derivative of arachidonic acid [12] and is formed by a variety of tissues through the 12-lipoxygenase/hepoxilin synthase pathways [13]. A recent report shows that HxA3 is produced by epithelial cells in response to bacterial infection [14]. Furthermore, HxA3 is a chemoattractant that is necessary and sufficient to recruit neutrophils to infected and inflamed sites [14, 15]. HxA3 causes the mobilization of intracellular calcium into the cytosol in neutrophils [16]. The increase in intracellular calcium causes activation of potassium current [17]. However, it is not known whether HxA3 can activate neutrophils to induce calcium dependent NETosis.

In this study, we sought to determine whether HxA3 can directly induce NETosis. We show that HxA3 and PBT-3, a synthetic analogue of HxB3, directly induce NETosis. These results were confirmed by plate reader assays as well as by immunofluorescence assays. Using DPI (20 *μ*M), a specific inhibitor of NADPH oxidase (NOX), we also demonstrate that HxA3-induced NETosis is NOX-dependent at lower concentrations of HxA3 but becomes NOX-independent at higher doses of HxA3. Therefore, HxA3 is a novel member of natural lipid NET-inducers.

## 2. Materials and Methods

### 2.1. Reagents

All reagents were purchased from Sigma unless otherwise stated.

### 2.2. Hepoxilin A3 and PBT-3

Both HxA3 and the Hx analogue, PBT-3, were produced through total chemical synthesis and used in this study as the methyl ester. HxA3 was provided by Corey and Su [18] and PBT-3 was synthesized in our laboratory by Demin and Pace-Asciak [19]. Stock solutions of the compounds were stored in benzene at −80°C. When required, aliquots were taken, the solvent was removed with a stream of N2 gas, and the residue was dissolved in DMSO at a concentration of 1 *μ*g/*μ*L for HxA3 and 2 *μ*g/*μ*L for PBT-3 and added to the cells.

### 2.3. Human Peripheral Neutrophils

Peripheral blood from healthy donors was collected in K2 EDTA blood collection tubes (BD, Franklin Lakes, NJ). A signed informed consent was obtained from each donor, and the protocol was approved by the Hospital for Sick Children ethics committee. Neutrophils were then isolated from whole blood using PolymorphPrep (Axis-Shield PoC, Oslo, Norway) according to the manufacturer's instructions with minor modifications. The modifications are as follows: (i) the lysis of red blood cells was done using a hypotonic saline solution (0.2% (w/v) NaCl) and was followed by the addition of an equal volume of 1.6% (w/v) NaCl solution with Hepes buffer (20 mM, pH 7.2) to make the solution buffered and isotonic. This was followed by two washes with a wash buffer containing 0.85% (w/v) NaCl and Hepes (10 mM, pH 7.2). Isolated neutrophils were then resuspended in RPMI (Invitrogen) supplemented with Hepes buffer (10 mM, pH 7.2). In order to assess the requirement of NADPH oxidase activity, neutrophils were preincubated with NOX inhibitor diphenyleneiodonium (DPI) (20 *μ*M) for 1 h prior to the activation of cells for NETosis. For each experiment, only 1-2 donor samples were processed each day. The assays were repeated with different donors to obtain experimental replicates.

### 2.4. Plate Reader Assay

For the plate reader assay, cells were seeded at 3 × 10^4^ cells per well in a 96-well plate in the culture media in the presence of Sytox Green cell impermeable nucleic acid stain (Life Technologies, Ontario, Canada) at 5 *μ*M. The fluorescence was measured using POLARstar OMEGA fluorescence microplate reader (BMG Labtech, Ortenberg, Germany) at specific time intervals for up to 240 min. To calculate the relative release of NET DNA, fluorescence readout obtained from cells lysed with 0.5% (v/v) Triton X-100 was considered as 100% DNA release, and the release of NET DNA at each time point was expressed as the % of total release.

### 2.5. Confocal Imaging

For imaging, cells used for the plate reader assay were fixed with PFA (4%, w/v) in PBS buffer for 10 min and permeablized with Triton X-100 (0.1%, w/v). Mouse-*α*-myeloperoxidase antibody (ab25989, Abcam, Ontario, Canada) at 1 : 500 dilution and an anti-mouse IgG secondary antibody conjugated with Alexa Fluor 555 (Invitrogen) were used for staining MPO. DNA was already stained with Sytox Green for the plate reader assay. The confocal images were taken using the Olympus IX81 inverted fluorescence microscope equipped with a Hamamatsu C9100-13 back-thinned EM-CCD camera and Yokogawa CSU X1 spinning disk confocal scan head (with Spectral Aurora Borealis upgrade) and 4 separate diode-pumped solid state laser lines (Spectral Applied Research, 405 nm, 491 nm, 561 nm, and 642 nm). The objectives used were 20 ×/ 0.75 or 60 ×/ 1.35. The microscope was operated with Volocity software (Perkin Elmer, Waltham, MA). Images taken on the spinning disk confocal microscope were deconvolved by iterative restoration with confidence limit set to 95% and iteration limit set to 20. All images were deconvolved to the confidence limit before reaching iteration limit.

### 2.6. Statistical Analysis

All data are presented as mean ± sem. Statistical analysis was performed using GraphPad Prism statistical analysis software (Version 5.0a for Mac OS X). Where appropriate, ANOVA with Bonferroni post-hoc test was used. A *P* value which was set at 0.05 or less was considered to be statistically significant.

## 3. Results and Discussion

### 3.1. Hepoxilin A3 Induces NETosis

A number of reports now suggest pathogenic roles for NETs in lung disorders [6, 7]. NETs have been shown to be associated with transfusion-related acute lung injury (TRALI) in an experimental mouse model, as well as in humans by two independent groups [6, 7]. In both of these studies, NETs were found to be present in circulation and in lungs [6, 7], and therapeutic strategies using DNase [6, 7] or anti-histone antibody [6] to target NETs were found to be protective. The damaging effect of NETs is thought to come directly from NETs, as it has been found that NETs are capable of inflicting injury to epithelial and endothelial cells [5, 20, 21].

**Figure 1:**
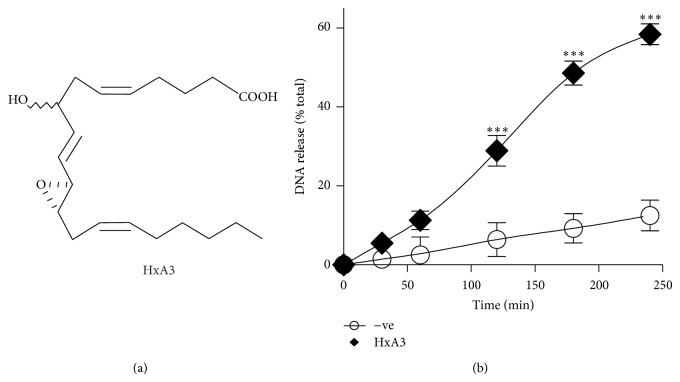
Hepoxilin A3 induces DNA release from human neutrophils. (a) Structure of HxA3. (b) Neutrophils were seeded into 96-well plates in the presence or absence of HxA3, and the extracellular DNA release was monitored using Sytox Green cell impermeable DNA dye. The results show a time-dependent increase in NET release after activation with HxA3 (10 *μ*g/mL) (*n* = 4). HxA3 was used as the methyl ester.

**Figure 2:**
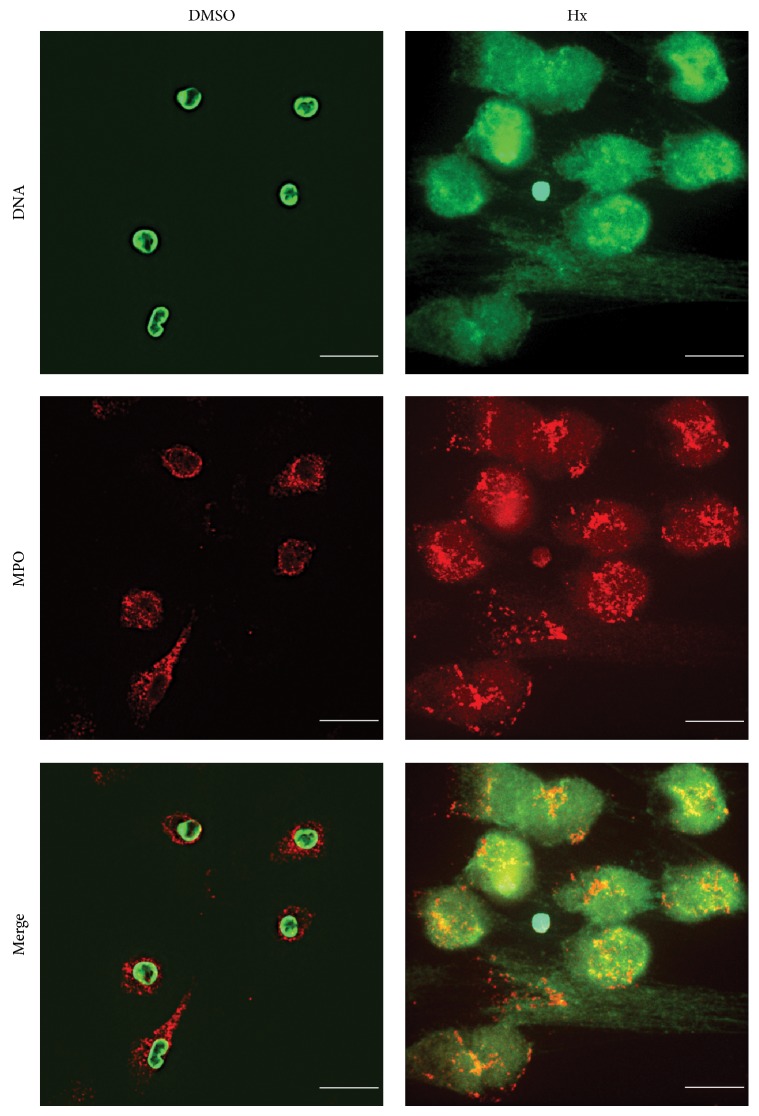
Hepoxilin A3 induces NETosis. Four hours after stimulation with either vehicle or HxA3, cells were fixed and stained for immunofluorescence analysis. Cells were stained for DNA (green) and MPO (red) after activation with HxA3 (5 *μ*g/mL). Images are representative of 3 independent experiments.

Here we sought to determine whether HxA3 directly induces NETosis in human neutrophils. HxA3 is an eicosanoid that acts as a lipid mediator of proinflammatory response [12, 15, 16]. In addition, our group has shown that HxA3 activates neutrophils and induces the release of intracellular calcium [16, 22]. Because calcium signaling is a critical component of NETosis, we asked whether HxA3 is a natural inducer of NETosis. The plate reader assays demonstrate a time-dependent NET release in response to HxA3 (10 *μ*g/mL; *P* < 0.001; Figure 1). Immunofluorescence imaging confirms that HxA3 induces NET formation and release and that HxA3-induced NETs contain myeloperoxidase (MPO) (Figure 2).

**Figure 3:**
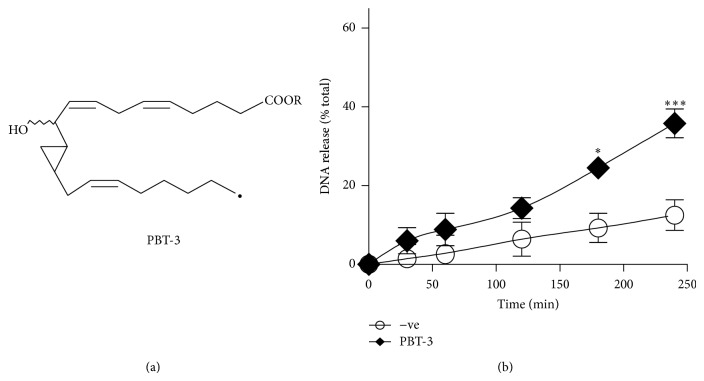
PBT-3 induces DNA release from human neutrophils. (a) Structure of PBT-3. (b) Neutrophils were seeded into 96-well plates in the presence or absence of PBT-3 (20 *μ*g/mL), and the extracellular DNA release was monitored using Sytox Green cell impermeable DNA dye. The results show a time-dependent increase in NET release after activation with PBT-3 (20 *μ*g/mL) (*n* = 4). R = Methyl.

**Figure 4:**
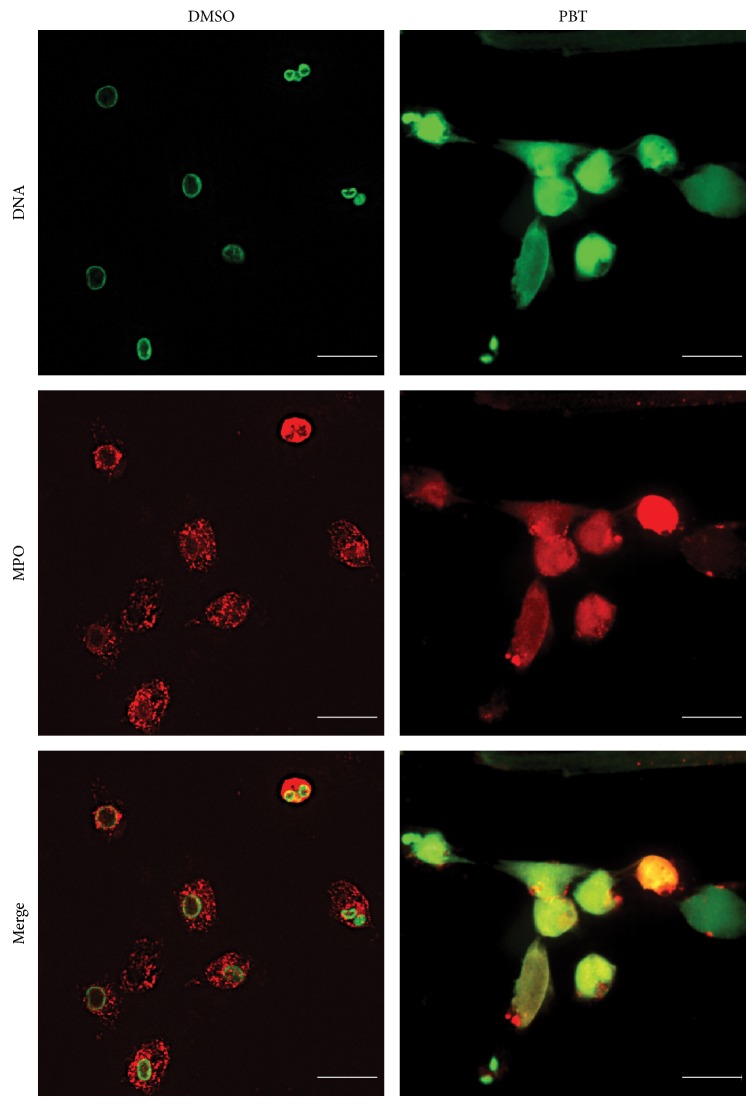
PBT-3 induces NETosis. Four hours after stimulation with either vehicle or PBT-3 (20 *μ*g/mL), cells were fixed and stained for immunofluorescence analysis. Cells were stained for DNA (green) and MPO (red) after activation with PBT-3 (20 *μ*g/mL). Images are representative of 3 independent experiments.

We next sought to confirm this finding by using PBT-3, a synthetic analogue of HxB3 [13, 19]. As expected, the plate reader assays demonstrate that NETs are released in response to PBT-3 (Figure 3). The plate reader assays also show that NET release by PBT-3 (20 *μ*g/mL) increases over time (*P* < 0.001); however, the extent of NET release was not as high as that induced by HxA3 at 240 min (Figure 1). This is not unexpected as PBT-3 is an analog of HxB3 (not HxA3), which is weak in releasing intracellular calcium from neutrophils. This strongly suggests that calcium plays an important role in mediating NETosis induced by HxA3. Immunofluorescence assays were then performed and confirmed that PBT-3 indeed induces NETosis as evidenced by the colocalization of extracellular DNA and MPO (Figure 4).

### 3.2. HxA3-Mediated NETosis Is Both NOX-Dependent and NOX-Independent Depending on HxA3 Concentration

**Figure 5:**
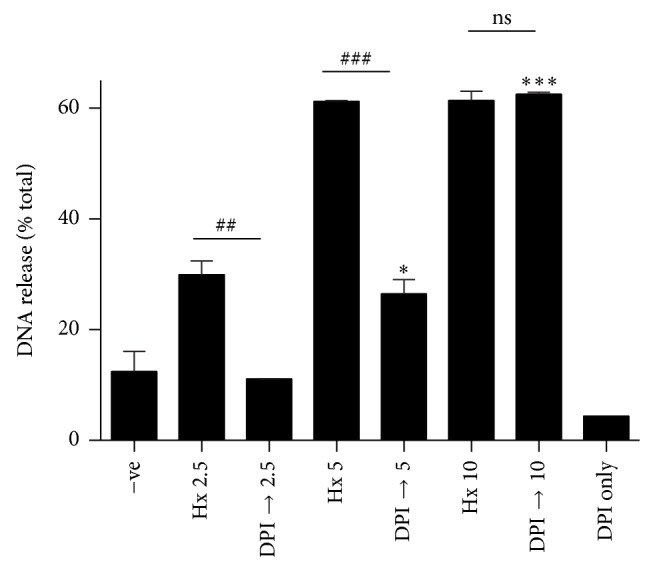
Hepoxilin A3-mediated NETosis is both NOX-dependent and NOX-independent depending on the dose of HxA3. A fluorescence plate reader assay was utilized to monitor NET DNA release upon stimulation with HxA3 in the presence or absence of the NOX inhibitor DPI (20 *μ*M) (*n* = 2; ^*^ *P* < 0.05; ^***^ *P* < 0.001 compared to cells activated with HxA3 (2.5 *μ*g/mL) in the presence of DPI (DPI → 2.5); ^##^ *P* < 0.01; ^###^ *P* < 0.001 (one-way ANOVA)).

We next asked whether HxA3-induced NETosis was NOX-dependent. The plate reader assays show that, at lower concentrations of HxA3 (2.5 and 5 *μ*g/mL), DPI significantly reduces NET release, suggesting that, at these concentrations, ROS generation by NOX is required (Figure 5). At higher concentrations of HxA3, there is less suppression of NETosis by DPI (*P* < 0.05; Figure 5). At the highest concentration tested (10 *μ*g/mL), HxA3-mediated NETosis completely overcomes the suppression by DPI and becomes NOX-independent (*P* < 0.001, Figure 5). These results suggest that HxA3-mediated NETosis has both NOX-dependent and NOX-independent features, conditioned by the HxA3 concentration.

Our group has shown previously that HxA3 activates neutrophils by inducing a rise in intracellular calcium concentration [16]. Furthermore, we have shown that the activation of neutrophils with HxA3 promotes the release of diacylglycerol (DAG) in addition to increasing intracellular calcium [23], pointing to the possibility that HxA3 activates PKC as well as calcium signaling. Of the two distinct forms of NETosis (i.e., NOX-dependent and NOX-independent pathways), calcium is indispensable in the NOX-independent pathway [24]. Furthermore, the activation of NOX-independent pathway in neutrophils results in a rapid release of NETs (<1 h) compared to the NOX dependent pathway (>2 h). Thus, it is possible that NOX-dependent and NOX-independent NETosis are conditioned by HxA3, whereby the activation of a specific pathway depends on HxA3 concentration.

As mentioned above, NETs have been shown to be involved in a number of inflammatory lung disorders [4]. Although some mechanistic insights have been obtained for lung disorders such as TRALI, VILI, and pneumonia [6–10], the precise mechanisms leading to abundant NET production in other conditions such as CF remain elusive. It has been shown that NETs are found in airways of patients with CF [11]. Bacterial pathogens such as* Pseudomonas aeruginosa* commonly infect CF airways. The overabundance of NET DNA can support the biofilm formation by* P. aeruginosa* [25–27]. Because hepoxilins can be produced by epithelial cells in response to* P. aeruginosa* infection [14], act as a chemoattractant for neutrophils [14, 15, 28], and directly induce NETosis (Figures 1 and 2), these compounds can be important therapeutic targets for NET-associated lung disorders.

## 4. Conclusions

The present study demonstrates that HxA3 can induce NETosis in human neutrophils. The results were corroborated by using PBT-3, a synthetic analogue of HxB3. The requirement for ROS generation by NOX in HxA3-mediated NETosis is dose-dependent, suggesting that HxA3 utilizes both NOX-dependent and NOX-independent pathways. Our finding that HxA3 directly induces NETosis suggests that HxA3 could induce NETosis in the lung, and hepoxilins and members of hepoxilin-generating pathways may therefore be therapeutic targets in NETs related inflammatory lung disorders.
